# Breast cancer incidence, stage, treatment and survival in ethnic groups in South East England

**DOI:** 10.1038/sj.bjc.6604852

**Published:** 2009-01-06

**Authors:** R H Jack, E A Davies, H Møller

**Affiliations:** 1 King's College London, Thames Cancer Registry, 1st Floor Capital House, 42 Weston Street, London SE1 3QD, UK

**Keywords:** ethnicity, breast cancer, incidence, stage, treatment, survival

## Abstract

Studies from the US have shown variations in breast cancer incidence, stage distribution, treatment and survival between ethnic groups. Data on 35 631 women diagnosed with breast cancer in South East England between 1998 and 2003 with self-assigned ethnicity information available were analysed. Results are reported for White, Indian, Pakistani, Bangladeshi, Black Caribbean, Black African and Chinese women. Age-standardised breast cancer incidence rate ratios, patterns of stage of disease at diagnosis, treatment, overall and breast cancer-specific survival were examined. All ethnic groups studied had lower age-standardised breast cancer incidence rates than White women, with Bangladeshi women having the lowest rate ratio (0.23, 95% CI: 0.20–0.26). White women were the most likely to have a stage recorded at diagnosis (adjusted proportion 75%), and least likely to be diagnosed with metastatic disease (7%). Black African women were the least likely to have a record of cancer surgery (63%) or hormone therapy (32%), and most likely to receive chemotherapy (38%). After fully adjusting for age, socioeconomic deprivation, stage of disease and treatment received, there was no significant variation in breast cancer-specific survival. However, Black African women had significantly worse overall survival (hazard ratio 1.24, *P*=0.025). These findings suggest that a strategy of earlier detection should be pursued in Black and South Asian women.

Data from the United States show a lower breast cancer incidence in Black than White women, (Ward *et al*, 2004; Smigal *et al*, 2006) even after adjusting for socioeconomic status (Chlebowski *et al*, 2005). Using country of birth and names to assign ethnicity, a significantly lower incidence has been found in South Asian than in non-South Asian women (Winter *et al*, 1999; Smith *et al*, 2003), and in women born in the ‘Caribbean Commonwealth’ and South Asia than in the general female population in England and Wales (Harding and Rosato, 1999).

Many comparisons of stage of disease at diagnosis and treatment of cancer patients by ethnic group have been made in the United States. Whereas Black (Li *et al*, 2003; Morris *et al*, 2007) and Indian/Pakistani women (Li *et al*, 2003) were more likely to have advanced stage disease than White women, Hahn *et al* (2007) found no association between ethnicity and advanced stage after adjustment for several factors. Black women in the US also appear less likely to receive systemic chemotherapy and hormone therapy (Naeim *et al*, 2006) and more likely to receive inappropriate treatment compared to national guidelines (Li *et al*, 2003) than White women. US studies have shown that Black women have a lower survival (Grann *et al*, 2006; Smigal *et al*, 2006), Chinese women have a better survival, and there is no difference between Indian/Pakistani women's survival compared with White women (Li *et al*, 2003).

The area of South East England covered by the Thames Cancer Registry (TCR) is the most ethnically diverse in England, including groups who are from low incidence areas of the world. Hospital Episode Statistics (HES) data are available for all inpatient admissions to English NHS hospitals and include self-assigned ethnicity, which is more complete than that in TCR (Jack *et al*, 2006). This study used all available ethnicity data to describe patterns of breast cancer incidence, stage, treatment and survival in different ethnic groups in South East England.

## Materials and methods

In the United Kingdom, cancer registries record the occurrence of cancer in their resident populations. In the area covered by the TCR, registration is initiated by clinical and pathological information received from hospitals and by information about deaths provided by the National Health Service Central Register through the Office for National Statistics. Trained data collection officers then extract further information on demographic details, disease stage and treatment in the first 6 months after diagnosis from the medical records. TCR uses a simple four-level staging system, using information in the patients' notes. This allows solid tumours to be assigned to categories based on whether the disease is local, has direct extension beyond the organ of origin, has regional lymph node involvement, or has metastasised. Data are quality assured as they are added to the central database. Hospital Episode Statistics data come from hospital Patient Administration Systems and include patient, clinical, administrative and geographical details. Self-assigned ethnicity was introduced to the HES data in April 1995 using the codes from the England and Wales 1991 Census. In April 2001 the new 2001 Census ethnic codes were brought in, although the 1991 codes were still accepted until March 2003.

Records on 55 710 women diagnosed with breast cancer (ICD-10 code C50) between 1998 and 2003 were extracted from the TCR database. Hospital Episode Statistics data on all patients residing in South East England with cancer (or suspected cancer) admitted to NHS hospitals between April 1997 and March 2004 were obtained. These patient records were matched to the TCR records using either NHS number, or sex, date of birth and postcode. The ethnicity code was extracted from HES and added to the TCR record. If there was no match, and an ethnic code was available from TCR records, this was used.

Ethnic groups were classified into the following categories: White, Mixed, Indian, Pakistani, Bangladeshi, Asian Other, Black Caribbean, Black African, Black Other, Chinese, Other or Not Known. Although analyses were performed using all available information, results are only presented for the seven groups that are easily combined from the 1991 and 2001 Censuses: White, Indian, Pakistani, Bangladeshi, Black Caribbean, Black African and Chinese. Socioeconomic deprivation was measured using the income domain of the Index of Multiple Deprivation 2000 (Department of the Environment, Transport and the Regions, 2000), divided into quintiles across England and Wales and assigned to records using postcode of residence at diagnosis.

It was not possible to determine an ethnic code for all patients. As there is no ‘ethnicity not known’ group in the population data based on the census, the absolute values of computed age-standardised incidence rates by ethnic group are too low due to the exclusion of the patients without a valid ethnic code. To address this, age-standardised rate ratios were calculated, using Census 2001 data as the population denominator, with White women as the baseline group. Confidence intervals were calculated using the method described in Boyle and Parkin (1991). These were also calculated separately for three truncated age groups: under 50, 50–64 and 65 years and older.

Patients who are registered by death certificate only have limited information recorded in the TCR database. These patients are included in the incidence analyses, but excluded from analyses on stage, treatment and mortality. There were 2117 (3.8%) cases registered by death certificate only in this dataset. Logistic regression was performed to analyse the availability of the patients' stage at diagnosis. If stage was recorded, logistic regression was again used to determine whether the patient was diagnosed with metastatic disease. Results from both analyses were transformed to generate proportions adjusted for age and socioeconomic deprivation. The proportions of each ethnic group receiving non-investigative cancer surgery, radiotherapy, chemotherapy, and hormone therapy within the first 6 months of diagnosis were calculated. Using logistic regression these proportions were adjusted for age at diagnosis, socioeconomic deprivation and stage at diagnosis.

Survival was analysed using Cox regression, with patients followed up until 31 December 2006. Deaths from all causes and from breast cancer were analysed separately. A death was considered to have been due to breast cancer if the ICD-10 code C50, ICD-9 code 174 or the word ‘breast’ were included in Part 1 of the death certificate. The estimated hazard ratios (HR) were sequentially adjusted for age, socioeconomic deprivation, stage at diagnosis and treatment received.

## Results

Ethnicity information was available for 35 631 (64%) of the 55 710 records of women diagnosed with breast cancer extracted from the database. Excluding cases registered by death certificate only, 34 998 (65%) of 53 593 patients had a known ethnicity. Incidence results are reported for the seven ethnic groups, which make up 60% (33 633) of the total cases. In all 33 024 (62%) cases had complete registration information rather than incomplete information from a death certificate only and the results for stage, treatment and survival analyses are shown for this group.

White women had the highest age-standardised breast cancer incidence rates. Incidence rate ratios were calculated with White women as the baseline group, and were all significantly below one: Indian (0.68; 95% CI, 0.64–0.73), Pakistani (0.59; 95% CI, 0.51–0.69), Bangladeshi (0.23; 95% CI, 0.20–0.26), Black Caribbean (0.80; 95% CI, 0.74–0.86), Black African (0.66; 95% CI, 0.59–0.74) and Chinese (0.54; 95% CI, 0.47–0.63).

**Figure 1:**
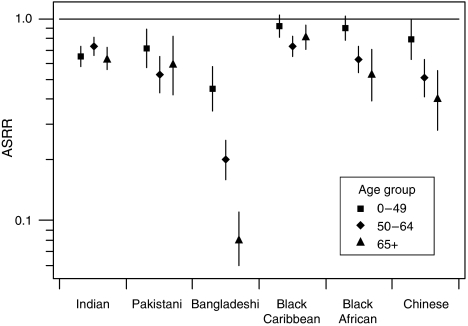
Age-standardised breast cancer incidence rate ratios (ASRR) and 95% confidence intervals by age group with White women as the baseline group.

Figure 1 shows age-standardised incidence rate ratios calculated separately for different age groups. Younger women of different ethnic groups had incidence rates more similar to White women of the same age. This pattern was evident in Bangladeshi, Black Caribbean, Black African and Chinese women, but was less clear for Pakistani women. There was no difference in the incidence rate ratios for the different age groups of Indian women.

Results of the analyses of stage, adjusted for age and socioeconomic deprivation, are shown in Table 1. White women were most likely to have a stage recorded at diagnosis (75%), and Bangladeshi women were least likely (55%). After excluding patients with stage not known, Pakistani women were most likely to be diagnosed with metastatic disease (17%) and White women the least likely (7%). White women were less likely to be diagnosed with metastatic disease than all other groups, although the difference was not significant for Bangladeshi and Chinese women due to small numbers.

Table 2 shows the proportions of each ethnic group receiving different treatments, adjusted for age, socioeconomic deprivation and stage. The largest differences were seen between White and Black African women. Black African women were less likely to have a record of cancer surgery (63 *vs* 72%, *P*=0.003) and hormone therapy (32 *vs* 54%, *P*<0.001), and more likely to receive chemotherapy (38 *vs* 29%, *P*=0.001) than White women. Pakistani women were significantly less likely to receive radiotherapy (27 *vs* 36%, *P*=0.043) and hormone therapy (41 *vs* 54%, *P*=0.014) than White women, whereas Black Caribbean women were less likely to receive hormone therapy (39 *vs* 54%, *P*<0.001). Several other associations were of borderline significance, or not significant because of the small numbers. Bangladeshi (41%, *P*=0.179), Chinese (47%, *P*=0.191) and Indian (50%, *P*=0.074) women were less likely to receive hormone therapy than White women (54%); Chinese (28 *vs* 36%, *P*=0.088) and Black Caribbean (32 *vs* 36%, *P*=0.078) were less likely to receive radiotherapy, and Chinese women were also more likely to receive surgery (79 *vs* 72%, *P*=0.148).

The patterns of survival using death from any cause are shown in Table 3, sequentially adjusted for age, socioeconomic deprivation, stage and treatment. Chinese women had a lower risk of dying than White women, which was largely unaffected by adjustment (fully adjusted HR=0.66, *P*=0.088). All other ethnic groups had higher risks of dying compared with White women after adjustment for age only. However, only Black African women's risk of dying remained high after additional adjustment for socioeconomic deprivation and stage (HR=1.42, *P*<0.001), and although attenuated, was still high after further adjustment for treatment (HR=1.24, *P*=0.025). Women living in the most socioeconomically deprived area had the worst overall survival (HR 1.25, *P*<0.001).

Results for breast cancer-specific survival, again adjusted for age, socioeconomic deprivation, stage and treatment, are shown in Table 4. There was a similar pattern to the overall survival: Chinese women had the lowest risk of dying from breast cancer (fully adjusted HR=0.63, *P*=0.089) and Black African women had the highest (fully adjusted HR=1.09, *P*=0.412). The worse breast cancer-specific survival in the other groups was attenuated by adjustment, and no significant association was found after adjusting for age, socioeconomic deprivation, stage and treatment.

## Discussion

This study found variations in breast cancer incidence, stage at diagnosis and treatment received between different ethnic groups in South East England. White women had the highest age-standardised incidence rates and Bangladeshi women the lowest. The rates in younger women from different ethnic groups appeared to be converging. White women were most likely to have a stage recorded at diagnosis and least likely to present with advanced stage disease. There was significant variation in the treatment received by different ethnic groups, but these differences were generally consistent with differences in the proportion of patients with advanced disease.

There was less variation in survival between ethnic groups. Chinese women had the best overall and breast cancer-specific survival. Black African women had the worst overall survival, but the effect was not as strong for breast cancer-specific survival. Variation among other ethnic groups was explained by adjustment for other variables. Although adjustment for stage and treatment explained the variation in breast cancer-specific survival, it did not explain the worse survival for women living in more deprived areas.

This study replicates previous findings that White women have higher incidence rates than Black women in the United States (Ward *et al*, 2004; Chlebowski *et al*, 2005; Smigal *et al*, 2006) and the United Kingdom (Harding and Rosato, 1999). It also confirms that South Asian women have lower incidence rates than White women (Harding and Rosato, 1999; Winter *et al*, 1999; Smith *et al*, 2003), and is able to verify this separately for Indian, Pakistani and Bangladeshi women.

Screening detects cancers which are asymptomatic and may not have otherwise been diagnosed or recorded. There is evidence that in the United Kingdom screening uptake is lower in women who live in more deprived areas (Maheswaran *et al*, 2006) and that White British people are less likely to live in the most deprived areas of England (Tinsley and Jacobs, 2006). The analyses of incidence in this study did not take deprivation into account and therefore the higher incidence rates in White women could be partly due to more screen-detected disease. Screening will also affect the stage of disease, as it will detect less advanced tumours before they become symptomatic. However, even after adjusting for socioeconomic deprivation, the results of this study are consistent with previous findings of more advanced disease at diagnosis in Indian, Pakistani and Black women (Li *et al*, 2003; Morris *et al*, 2007). Hahn *et al* (2007) found no difference in disease stage at diagnosis between Black and White women after adjustment for several factors, including age, level of education, insurance status, poverty, method of detection and tumour characteristics. It is possible that some of these factors would explain the variation found in the present study.

In the United States, Black women are less likely to receive chemotherapy and hormone therapy (Naeim *et al*, 2006). In our study, Black Caribbean and Black African women were less likely to receive hormone therapy, but the latter were more likely to receive chemotherapy. This pattern of treatment in Black African women may reflect differences in stage and triple-negative disease (oestrogen receptor, progesterone receptor and human epidermal growth factor receptor 2 (HER2) negative). Studies in the United States have shown that Black women are more likely to have triple-negative breast cancer than White women (Bauer *et al*, 2007; Morris *et al*, 2007). This was also suggested by a study of patients below age 60 in a hospital population in London, although this result was not significant (Bowen *et al*, 2008). Triple-negative disease does not respond to hormone therapy, and chemotherapy may be the preferred treatment option for these cancers (Cleator *et al*, 2007).

US Black women had worse survival than White women, even after accounting for patient, tumour and geographical factors (Li *et al*, 2003; Grann *et al*, 2006). Chinese women had better survival than White women, and there was no significant difference between Indian/Pakistani and White women after adjusting for age, tumour characteristics and treatment (Li *et al*, 2003). An earlier study in the TCR area found women with South Asian names had better relative survival than non-South Asian women (dos Santos Silva *et al*, 2003). In a London hospital population Black women with smaller tumours had significantly worse overall survival than White women with similar sized tumours, adjusting for age and socioeconomic deprivation (Bowen *et al* 2008). In our study, Black African women had significantly worse overall survival after adjustment for age, socioeconomic deprivation, stage at diagnosis and treatment, and Chinese women had better survival, although not significantly so due to small numbers. No significant variation in survival was found in Indian, Pakistani or Bangladeshi women.

A significant limitation of this study is that ethnicity information was not available for a large proportion (36%) of the patients. This could potentially bias the results if particular ethnic groups were over or underrepresented in the missing data. The computed incidence rate ratios assume that the proportion of missing values was the same in each of the different ethnic groups. All women diagnosed, including those with unknown ethnicity, were included in the analyses for stage, treatment and survival. Women with unknown ethnicity had a lower risk of dying from all causes (fully adjusted HR=0.89, *P*<0.001) and from breast cancer (HR=0.90, *P*<0.001) than White women. A sensitivity analysis was performed using the extreme assumption that all women of unknown ethnicity were White. The results for the other ethnic groups compared with this extended White group were very similar to the ones presented. Black African women had a significantly high fully adjusted HR when using all causes of death (HR=1.29, *P*=0.009), and although their breast cancer-specific survival was also high, it was not statistically significant (HR=1.13, *P*=0.252). All other ethnic groups had non-significant HRs compared with the new group of White women and women with unknown ethnicity. Improving ethnicity data collection is a key target for routinely collected datasets including hospital records, HES, and cancer registers.

Although this study was able to investigate ethnic groups at a more detailed level than most previous studies, there could still be important differences within these groups. For example, McCormack *et al* (2004) examined groups defined by place of birth (including area within country), first language and religion. The associations between these groups and risk of breast cancer lost their significance after adjustment for reproductive, socioeconomic, anthropometric and dietary factors. These and other factors such as comorbidity, which are not routinely recorded by cancer registries could contribute to the differences in treatment and survival. As the registry only collects information on treatment received up to 6 months after diagnosis, some treatment data are missing, although this is unlikely to vary by ethnic group.

The interpretation of survival differences between ethnic groups must recognise the likelihood of strong associations between socioeconomic factors, disease stage at diagnosis and treatment. In the analysis of breast cancer-specific survival, there was a gradual attenuation of the survival differences between ethnic groups as more variables were adjusted for. The adjustment for socioeconomic deprivation, disease stage and treatment all tended to work towards a reduction of the excess mortality in the South Asian and Black groups. The most plausible interpretation is that survival differences are principally due to variation in stage at presentation, which in these data is manifest both through the recorded stage at diagnosis, but also independently through the recorded socioeconomic deprivation and the administered treatment. It seems very unlikely that there are biologically based differences between the ethnic groups, which confer strong survival differences, independently of stage and treatment. The only subanalysis that suggests this is the fully adjusted analysis of overall survival in Black African women where the HR persisted at a statistically significant value of 1.24. However, this estimate was highly sensitive to adjustment for deprivation, stage and treatment, which leaves room for an effect of residual confounding. Further, the corresponding estimate in the breast cancer-specific analysis was much lower at 1.09. Therefore, the present data suggest that breast cancer services may usefully pursue a strategy of earlier detection with the aim of achieving a more favourable stage distribution at diagnosis. This should be a particular priority for the South Asian and Black groups analysed here.

To determine more robustly whether biological, behavioural or clinical factors are driving the observed differences, more information is needed on patient characteristics. The most important component is disease stage at diagnosis, as this will affect both the treatment received and survival. Improving the completeness of staging information as well as ethnicity information received by cancer registries will enhance future analyses. More advanced stage at diagnosis could reflect a more aggressive form of disease, later presentation because of a lack of awareness about symptoms or a mistrust of the health system, lower uptake of screening or delays in diagnosis. There is evidence that different ethnic groups have different ideas about causes of and candidacy for breast cancer (Pfeffer, 2004), which may affect attitudes towards accessing healthcare services. A better understanding of these beliefs would therefore be important to guide a strategy for earlier detection. Studies in the United States have also shown that Black women are more likely to have high risk tumour characteristics (Elledge *et al*, 1994) that are associated with earlier death from breast cancer (Anderson *et al*, 2005). Earlier detection is likely to be particularly important for these patients.

Although there are differences in recorded stage of disease at diagnosis and treatment between ethnic groups, it is encouraging that there is little variation in survival after adjustment for these factors, suggesting that some of the survival differences found in previous US studies (Grann *et al*, 2006; Smigal *et al*, 2006) may reflect the problems some groups have in accessing effective treatments in US healthcare systems. With better access to earlier detection, and the possibility of more aggressive treatment, survival could be improved for all women.

## Figures and Tables

**Table 1:** Percentage of breast cancer patients having a stage recorded at diagnosis, and of staged patients, those with metastatic disease at diagnosis by ethnic group, adjusted for age and socioeconomic deprivation

		**Staged**			**Metastatic disease**		
	**Total**	***n***	**%**	***P*-value**	***n***	**%**	***P*-value**
White	31 109	23 271	75		1743	7	
Indian	639	446	67	<0.001	43	11	0.012
Pakistani	121	86	69	0.128	13	17	0.003
Bangladeshi	42	25	55	0.007	3	13	0.297
Black Caribbean	652	493	73	0.270	57	11	0.003
Black African	344	253	71	0.082	36	15	<0.001
Chinese	117	85	71	0.388	7	10	0.435

**Table 2:** Percentage of breast cancer patients receiving different treatments by ethnic group, adjusted for age, socioeconomic deprivation and stage

		**Cancer surgery**			**Radiotherapy**			**Chemotherapy**			**Hormone therapy**		
	**Total**	***n***	**%**	***P*-value**	***n***	**%**	***P*-value**	***n***	**%**	***P*-value**	***n***	**%**	***P*-value**
White	31 109	22 421	72		11 060	36		9006	29		16 678	54	
Indian	639	469	70	0.398	231	35	0.955	229	26	0.185	275	50	0.074
Pakistani	121	91	75	0.584	32	27	0.043	51	30	0.749	40	41	0.014
Bangladeshi	42	29	69	0.706	13	36	0.949	19	27	0.770	11	41	0.179
Black Caribbean	652	492	73	0.712	199	32	0.078	276	32	0.156	203	39	<0.001
Black African	344	234	63	0.003	100	33	0.390	205	38	0.001	70	32	<0.001
Chinese	117	95	79	0.148	31	28	0.088	58	30	0.834	43	47	0.191

**Table 3:** Hazard ratios for all cause mortality in breast cancer patients diagnosed 1998–2003

	**Adjusted for age**				**Socioeconomic deprivation**				**Stage**				**Treatment**			
	**HR**	**95%**	**CI**	***P*-value**	**HR**	**95%**	**CI**	***P*-value**	**HR**	**95%**	**CI**	***P*-value**	**HR**	**95%**	**CI**	***P*-value**
*Ethnic group*																
White	1.00				1.00				1.00				1.00			
Indian	1.10	0.93	1.29	0.256	1.05	0.89	1.23	0.590	0.92	0.78	1.08	0.299	0.91	0.78	1.08	0.285
Pakistani	1.22	0.85	1.76	0.278	1.14	0.79	1.64	0.493	0.95	0.66	1.36	0.766	0.92	0.64	1.33	0.665
Bangladeshi	1.08	0.56	2.08	0.818	0.99	0.51	1.90	0.972	0.76	0.39	1.46	0.406	0.88	0.46	1.69	0.695
Black Caribbean	1.23	1.06	1.43	0.006	1.11	0.96	1.29	0.157	1.01	0.87	1.18	0.864	0.98	0.85	1.14	0.811
Black African	1.83	1.51	2.21	<0.001	1.64	1.36	1.99	<0.001	1.42	1.18	1.72	<0.001	1.24	1.03	1.50	0.025
Chinese	0.69	0.43	1.11	0.123	0.67	0.42	1.08	0.104	0.66	0.41	1.07	0.089	0.66	0.41	1.06	0.088
Test for heterogeneity: *χ*^2^ (6 d.f.)	50.5			*P*<0.0001	31.5			*P*<0.0001	18.4			*P*=0.0053	9.7			*P*=0.1383
*Socioeconomic deprivation*																
1 (most affluent)					1.00				1.00				1.00			
2					1.06	1.01	1.11	0.027	1.09	1.04	1.15	<0.001	1.10	1.04	1.15	<0.001
3					1.17	1.11	1.23	<0.001	1.19	1.13	1.25	<0.001	1.19	1.13	1.25	<0.001
4					1.22	1.16	1.28	<0.001	1.25	1.19	1.31	<0.001	1.24	1.18	1.30	<0.001
5 (most deprived)					1.36	1.29	1.43	<0.001	1.27	1.21	1.34	<0.001	1.25	1.19	1.32	<0.001
Test for trend: *χ*^2^ (1 d.f.)					169.9			*P*<0.0001	111.3			*P*<0.0001	96.9			*P*<0.0001
*Treatment*																
Cancer surgery													0.49	0.48	0.51	<0.001
Radiotherapy													0.78	0.75	0.81	<0.001
Chemotherapy													1.53	1.46	1.60	<0.001
Hormone therapy													0.90	0.87	0.93	<0.001

**Table 4:** Hazard ratios for breast cancer mortality (coded cause 1a, 1b or 1c mentioning ‘C50’ or ‘174’ or text cause 1a, 1b or 1c mentioning ‘breast’) in breast cancer patients diagnosed 1998–2003

	**Adjusted for age**				**Socioeconomic deprivation**				**Stage**				**Treatment**			
	**HR**	**95%**	**CI**	***P***	**HR**	**95%**	**CI**	***P***	**HR**	**95%**	**CI**	***P***	**HR**	**95%**	**CI**	***P***
*Ethnic group*																
White	1.00				1.00				1.00				1.00			
Indian	1.08	0.89	1.30	0.437	1.02	0.85	1.23	0.809	0.86	0.71	1.03	0.105	0.86	0.71	1.04	0.118
Pakistani	1.19	0.79	1.80	0.400	1.11	0.74	1.67	0.625	0.87	0.58	1.31	0.496	0.87	0.58	1.31	0.501
Bangladeshi	1.25	0.65	2.40	0.507	1.13	0.59	2.18	0.711	0.85	0.44	1.63	0.618	1.00	0.52	1.93	0.990
Black Caribbean	1.31	1.11	1.55	0.001	1.18	1.00	1.39	0.054	1.03	0.87	1.21	0.748	1.01	0.85	1.19	0.953
Black African	1.74	1.42	2.14	<0.001	1.56	1.27	1.91	<0.001	1.27	1.03	1.56	0.025	1.09	0.89	1.34	0.412
Chinese	0.67	0.39	1.13	0.131	0.65	0.39	1.10	0.112	0.62	0.37	1.06	0.079	0.63	0.38	1.07	0.089
Test for heterogeneity: *χ*^2^ (6 d.f.)	41.6			*P*<0.0001	23.8			*P*=0.0006	11.8			*P*=0.0657	6.5			*P*=0.3679
																
*Socioeconomic deprivation*																
1 (most affluent)					1.00				1.00				1.00			
2					1.10	1.03	1.17	0.004	1.14	1.07	1.21	<0.001	1.13	1.07	1.21	<0.001
3					1.19	1.12	1.27	<0.001	1.21	1.14	1.29	<0.001	1.21	1.14	1.29	<0.001
4					1.24	1.16	1.32	<0.001	1.26	1.18	1.34	<0.001	1.25	1.17	1.33	<0.001
5 (most deprived)					1.41	1.33	1.51	<0.001	1.26	1.18	1.35	<0.001	1.27	1.19	1.35	<0.001
Test for trend: *χ*^2^ (1 d.f.)					124.7			*P*<0.0001	61.6			*P*<0.0001	62.0			*P*<0.0001
																
*Treatment*																
Cancer surgery													0.47	0.45	0.49	<0.001
Radiotherapy													0.85	0.82	0.89	<0.001
Chemotherapy													1.76	1.67	1.85	<0.001
Hormone therapy													0.86	0.83	0.90	<0.001
